# The Transcultural Diabetes Nutrition Algorithm: A Canadian Perspective

**DOI:** 10.1155/2014/151068

**Published:** 2014-01-16

**Authors:** Réjeanne Gougeon, John L. Sievenpiper, David Jenkins, Jean-François Yale, Rhonda Bell, Jean-Pierre Després, Thomas P. P. Ransom, Kathryn Camelon, John Dupre, Cyril Kendall, Refaat A. Hegazi, Albert Marchetti, Osama Hamdy, Jeffrey I. Mechanick

**Affiliations:** ^1^Crabtree Nutrition Laboratories, McGill University Health Centre/Royal Victoria Hospital, Montreal, QC, Canada H3H 1A1; ^2^Crabtree Nutrition Laboratories, McGill University Health Centre/Royal Victoria Hospital H6.90, 687 Pine Avenue West, Montreal, QC, Canada H3A 1A1; ^3^Department of Pathology and Molecular Medicine, Faculty of Health Sciences, McMaster University, Hamilton, ON, Canada L8N 3Z5; ^4^Toronto 3D Knowledge Synthesis and Clinical Trials Unit, Clinical Nutrition and Risk Factor Modification Centre, St. Michael's Hospital, Toronto, ON, Canada M5C 2T2; ^5^Department of Nutritional Sciences and Medicine, Faculty of Medicine, University of Toronto, Toronto, ON, Canada M5S 3E2; ^6^Clinical Nutrition and Risk Factor Modification Center, Division of Endocrinology and Metabolism, and Li Ka Shing Knowledge Institute of St. Michael's Hospital, Toronto, ON, Canada M5C 2T2; ^7^McGill University Health Centre/Royal Victoria Hospital, Montreal, QC, Canada H3A 1A1; ^8^Division of Human Nutrition, Division of Agriculture, Food and Nutritional Science, and the Alberta Diabetes Institute, University of Alberta, Edmonton, AB, Canada T6G 2E1; ^9^Centre de Recherche de L'Institut Universitaire de Cardiologie et de Pneumologie de Québec, Québec, QC, Canada; ^10^Department of Kinesiology, Faculty of Medicine, Université Laval, Québec, QC, Canada G1V 4G5; ^11^Division of Endocrinology and Metabolism, Capital Health, Halifax, NS, Canada B3H 2Y9; ^12^Dalhousie University, Canada; ^13^Department of Allied Health, Clinical Nutrition, University Health Network, Toronto, ON, Canada M5G 2C4; ^14^Robarts Research, University of Western Ontario, London, ON, Canada N6A 5B7; ^15^Clinical Nutrition and Risk Factor Modification Center, St Michael's Hospital, Toronto, ON, Canada M5S 3E2; ^16^Department of Nutritional Sciences, Faculty of Medicine, University of Toronto, Toronto, ON, Canada; ^17^College of Pharmacy and Nutrition, University of Saskatchewan, Saskatoon, SK, Canada; ^18^Abbott Laboratories, Abbott Park, 43219, USA; ^19^Medical Education and Research Alliance (Med-ERA), New York, NY 10019, USA; ^20^Department of Preventive Medicine and Community Health, University of Medicine and Dentistry of New Jersey, Newark, NJ 07101, USA; ^21^Joslin Diabetes Center, Harvard University, Boston, MA 02215, USA; ^22^Division of Endocrinology, Diabetes, and Bone Disease, Icahn School of Medicine at Mount Sinai, New York, NY 10029, USA

## Abstract

The Transcultural Diabetes Nutrition Algorithm (tDNA) is a clinical tool designed to facilitate implementation of therapeutic lifestyle recommendations for people with or at risk for type 2 diabetes. Cultural adaptation of evidence-based clinical practice guidelines (CPG) recommendations is essential to address varied patient populations within and among diverse regions worldwide. The Canadian version of tDNA supports and targets behavioural changes to improve nutritional quality and to promote regular daily physical activity consistent with Canadian Diabetes Association CPG, as well as channelling the concomitant management of obesity, hypertension, dyslipidemia, and dysglycaemia in primary care. Assessing glycaemic index (GI) (the ranking of foods by effects on postprandial blood glucose levels) and glycaemic load (GL) (the product of mean GI and the total carbohydrate content of a meal) will be a central part of the Canadian tDNA and complement nutrition therapy by facilitating glycaemic control using specific food selections. This component can also enhance other metabolic interventions, such as reducing the need for antihyperglycaemic medication and improving the effectiveness of weight loss programs. This tDNA strategy will be adapted to the cultural specificities of the Canadian population and incorporated into the tDNA validation methodology.

## 1. Introduction

**Figure 1:**
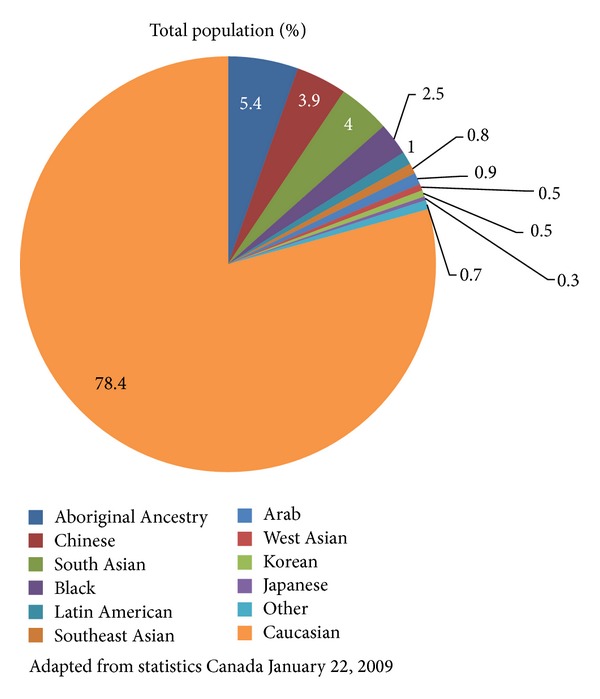
Canadian Population Demographics.

Type 2 diabetes (T2D) is a chronic disease with hyperglycaemia as its characteristic feature, resulting from defects in insulin secretion and/or insulin action [1]. The disorder is associated with adiposity, particularly central abdominal adiposity [2], and multiple metabolic abnormalities that increase the risk of mortality from cardiovascular diseases (CVD) by two- to fourfold [3], the leading cause of death [4], shortening life by 5 to 15 years. In Canada, the prevalence of T2D is increasing at epidemic proportions, affecting more than three million Canadians, with 6 million others at elevated risk of developing the disease. Of particular concern are non-Caucasians who comprise more than 25% of the Canadian population (Figure 1) and are highly susceptible to T2D when adopting a Western lifestyle. Diabetes affects economic prosperity, costing the Canadian healthcare system $12.2 billion annually, a number that is projected to rise to $16.9 billion by 2020 [4].

Canadian census data show that more than 200 tongues are spoken in Canada, 60 being aboriginal. The mother tongue reported by 6.8 million Canadians (21% of the population) differs from English or French, the two official languages of the country. Another 4.7 million Canadians speak a language at home by order of prevalence: Punjabi, Chinese, Spanish, Italian, German, Cantonese, Tagalog, Arabic, and Mandarin (Statistic Canada). Challenged by this situation, the Canadian Diabetes Association (CDA) has begun to tailor its nutrition therapy tool, Just the Basics, to the cultural and personal tastes of individuals of varied ethnicities who have T2D (http://www.diabetes.ca/diabetes-and-you/nutrition/just-basics/Accessed January 24, 2013).

Through the support of the CDA, clinical practice guidelines (CPG) for nutrition therapy [4] were published in 2003 and 2008 and updated in 2013, to provide evidence-based recommendations for healthy food choices and lifestyles that improve glycaemic, metabolic, and weight control. Included in these guidelines is the recommendation to replace high-glycaemic index (GI) carbohydrate foods by low GI carbohydrate foods in mixed meals because low GI intake is associated with a lower glycaemic response and improvements in A1C [4]. A multitude of cultures and diverse geographic locations across Canada have challenged the effectiveness of nutrition therapy guidelines to promote sustained healthy eating habits in the diabetic population. In response, Mechanick et al. [5] have designed a global tDNA template for the optimization of nutritional care in prediabetes and T2D on a global scale with the intention that provided information will suit geographic and ethnocultural factors for individualization and implementation at regional and local levels worldwide. It is anticipated that tDNA will increase awareness of the benefits of dietary behaviour changes, which can be better achieved when recommended dietary patterns and food choices accommodate regional differences in genetic factors, food availability and preferences, lifestyles, and cultures. Thereafter, a task force was selected among Canadian health care experts in diabetes and nutrition to adapt the global tDNA template to Canadian mores, norms and population demographics (Figure 1). These experts, who are authors of this paper, are also key regional stakeholders in the implementation of the CDA CPG.

## 2. Methods

The process of modifying the tDNA to Canada involved a group of experts who reviewed and considered revising all of the topics outlined in the global template [5]. These reviewers also defined a vision for the Canadian tDNA and considered factors unique to the Canadian population and the guidelines and recommendations put forth by CDA in their revision, that is, ethnocultural lifestyle input; individual risk stratification with tables on classification by body composition; general recommendations on physical activity and healthy eating, with the related tables providing physical activity and nutritional guidelines; specific recommendations for obesity, hypertension, and dyslipidemia; criteria for bariatric surgery; description of an antihypertensive diet and other dietary patterns; and the glyceamic indices and load of common foods. During development of the Canadian version of tDNA, the task force established that their shared vision of the tDNA was to enable sustainable healthy lifestyle behaviours amongst healthcare providers and people with diabetes. It became evident that primary care providers would best be implicated in promoting a healthy lifestyle in their patients with T2D if they believed in its positive impact on glycaemic and metabolic control to the point that they themselves adapt and sustain a healthy lifestyle, the latter made achievable through resources and tools within the Canadian tDNA. There was also a need for defining simple assessment measures for lifestyle behaviour that put Canadians at risk of developing T2D, or associated complications, and also measures of lifestyle behavioural change.

Changes brought to the global tDNA template and different points of significance are highlighted in the results section of this report.

## 3. Results

**Figure 2:**
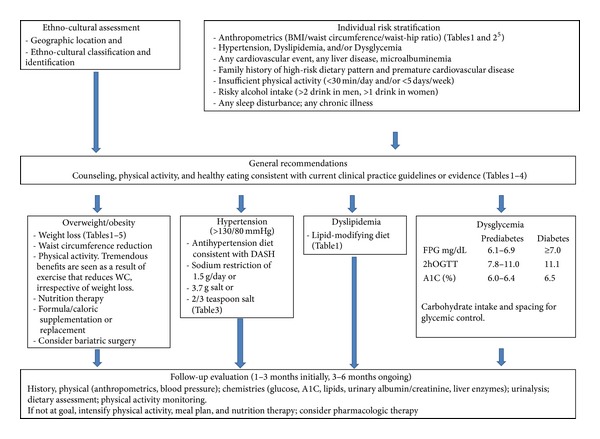
Canadian Transcultural Diabetes Nutrition Algorithm (tDNA) for prediabetes and type 2 diabetes.

As a result of our revision, the modified algorithm focused on the process of adapting healthy behaviours rather than weight loss; behaviour leading to improved diet and regular physical activity became the interventional target. Emphasis was placed also on increasing patient and provider awareness of the process of change and of improvements to lifestyle behaviour over time. The resultant objective of tDNA Canada became support of behavioural change through simple and effective dietary and physical activity advice at the primary care level. Additionally, a quick, simple, pragmatic, validated questionnaire to assess combined work and leisure time physical activity shown to be associated with mortality in a prospective population study was found to be feasible for use in clinical practice [6]. The algorithm was modified to better assist physicians in improving lifestyle habits of Canadian patients who present with diverse ethnic backgrounds, based on the existing Canadian guidelines for prevention and treatment of T2D. The global tDNA [5] adapted to fit current Canadian guidelines is presented in Figure 2.

As in the global tDNA, initially, ethnooncultural identification and geographic location are assessed concurrently with individual risk stratification, the latter described by Yusuf et al. [3]. In Canada, however, the general recommendations for counselling on care, physical activity, and healthy eating habits conform to CDA 2013 CPG [4] and are for all patients independent of the magnitude of their risk. Furthermore, the recommendations are extended to address patients' obesity, hypertension, dyslipidemia, and/or dysglycaemia with specific dietary approaches and metabolic targets. In all cases, follow-up evaluation is recommended at 1–3 months initially and at 3–6 months ongoing. The Canadian tDNA, like other cultural versions, includes and refers to tables that convey additional information adapted according to national or regional recommendations, in this case, Canadian CPGs. Examples are given in Tables 1–5.

Specific to the Canadian tDNA, Table 1 presents diabetes nutrition therapy in a manner that facilitates the selection of a strategy based on individualized targeted outcomes. Different dietary patterns evaluated in T2D populations, popular weight loss approaches, specific foods, varied macronutrient distributions, and meal replacements are listed with their specific effects on hemoglobin A1c (A1C), weight, blood pressure, lipid risk factors, inflammatory markers, hypoglycemia, and other advantages and disadvantages related to their impact on nutrients, gastrointestinal tract, or renal load. This approach is also promoted in the CDA CPGs [4].

Certain approaches from the global tDNA [5] were adapted to the many ethnicities in the Canadian population.  Table 2 is an example that shows a list of common foods and their GI. Other modifications to the global tDNA were based on the recommendations from CDA CPG [4]. For example, Table 3 summarizes those for physical activity in the management of diabetes [4] and Table 4 the Dietary Approaches to Stop Hypertension (DASH) in diabetes adapted according to CDA CPG.  Table 5 summarizes CDA CPG for bariatric surgery, which may be considered in patients with T2D and a BMI ≥ 35 kg/m^2^ when lifestyle interventions have failed to achieve and maintain weight goals. Minimally invasive surgical approaches should be used by a well-established surgical team that includes experts in nutritional and psychological support. Bariatric surgery is now becoming an accepted option for the management of T2D and has been shown to be superior to medical management for its treatment [6]. Presently there is no overall consensus as to what kind of procedure is most effective, be it malabsorptive, restrictive, or combination surgery.

## 4. Discussion

Adopting healthy behaviour rather than only attaining sustained weight loss was defined as the main objective of the Canadian tDNA. This objective is consistent with CDA CPG, which recommend that self-management education incorporating knowledge and skill development, as well as cognitive behavioural interventions, should be implemented for people with diabetes (CDA CPG 2008) [4]. To optimize change, messages regarding nutrition recommendations and lifestyle modification should accommodate a person's culture [7]. The ethnic mosaic of the Canadian population provides a rich testing ground for learning how to adapt educational tools to various cultures. At this time, the dietary education tool, Just the Basics, has been created for South Asian, Latin American, and the Aboriginal communities in Canada, using a consultative process within the respective cultural groups [8]. These tools were developed by reaching out to the ethnic communities through professional and community group networks to identify persons who could contribute to the adaptation of the educational materials. People with diabetes and their family members, as well as Aboriginal, Latin American, or South Asian dietitians, other dietitians experienced in working with cultural groups, and an advisory group of dietitians with expertise in diabetes management, participated in focus group discussions and pilot-tested the tools. The focus groups explored topics such as dietary patterns practiced in Canada, meaningful expressions of portion sizes, cultural holidays and values, preferred teaching and learning methods, classification of foods into food groups, and barriers to education [9]. Just the Basics provides clear initial messages to patients about healthy eating and physical activity for diabetes prevention and management, using culturally distinct foods and languages. Although these culturally adapted tools for the South Asian, Latin American, and Aboriginal populations need ongoing assessment of their ability to promote sustained behaviour change associated with optimal diabetes control, they provide the groundwork for creating tools tailored to other high-risk populations.

The Canadian tDNA is to promote low-GI carbohydrate foods within a healthy dietary pattern. GI provides an assessment of the quality of carbohydrate-containing foods based on their effect on postprandial blood glucose [10]. To decrease the glycaemic response to dietary intake, low-GI carbohydrate foods can replace high-GI carbohydrate foods. More detailed lists can be found in the International Tables of Glycaemic Index and Glycaemic Load Values [11]. Meta-analyses of controlled dietary trials of replacing high-GI carbohydrates with low-GI carbohydrates in the context of mixed meals have shown clinically significant improvements in glycaemic control over 2 weeks to 6 months in people with type 1 diabetes (T1D) or T2D [12–14]. Replacing high-GI carbohydrates with low-GI carbohydrates in mixed meals also has been shown to reduce total cholesterol over 2 to 24 weeks in people with and without diabetes [13], postprandial glycaemia and high-sensitivity C-reactive protein (hsCRP) over 1 year in people with T2D [15], and the number of hypoglycaemic events over 24 to 52 weeks in adults and children with T1D [14]. Similar benefits have been shown when low-GI diets are compared with different control diets. Dietary advice to consume a low-GI diet compared with a high-cereal fibre diet in people with T2D has been shown to improve glycaemic control and HDL cholesterol over 6 months [16]. In another trial in which dietary pulses (e.g., beans, chickpeas, lentils, and peas) were emphasized to lower the GI of the diet, significant improvements in glycaemic control and blood pressure were reported over 3 months [17]. A low-GI diet compared with a low-carbohydrate, high mono-unsaturated fat diet, has been shown to improve beta-cell function over one year in people with T2D [18]. Moreover, low-GI diets compared with dietary advice based on the nutrition recommendations of varied diabetes associations have been shown to have advantages. For example, (a) dietary advice to consume a low-GI diet improved glycaemic control over 3 months in Japanese people with impaired glucose tolerance (IGT) or T2D when compared with the nutritional recommendations of the Japanese Diabetes Society [19], and (b) the need for antihyperglycaemic medications over one year was decreased in people with poorly controlled T2D when compared with the nutritional recommendations of the American Diabetes Association [20].

The product of mean GI and total carbohydrate intake is known as glycaemic load (GL) and has also been explored in therapeutic studies. A low GL was found to improve the efficiency of weight loss advice over 4 weeks [21] and improve risk factors for coronary heart disease including high-density lipoprotein cholesterol (HDL-C), triglycerides, and C-reactive protein (CRP) over 4 weeks to 6 months [21–23] compared with a low-fat diet, in young overweight and obese adults without diabetes. A low GL diet has also been shown to have advantages for coronary heart disease in a systematic review and meta-analysis of prospective cohort studies [24] and for diabetes management itself in different analyses of the Nurses Health Study [25, 26]. The success of weight loss strategies using low-GL diets appears to be related to the degree of insulin resistance as assessed by the 30-min postprandial insulin loads [23].

The Canadian tDNA integrates and emphasizes physical activity. The recommendations are based on evidence from prospective observational studies showing that individuals who perform such levels of activity have reduced risk of premature total and cardiovascular mortality as well as reduced risk of developing T2D [27–30]. The relationship between level of physical activity and mortality/morbidity is semi-independent from the concomitant influence of well-established CVD risk factors such as lipids, blood pressure, diabetes, and smoking [28, 31]. Thus, even among individuals who are abdominally obese with other features of the metabolic syndrome, those who reported being very active exhibit a 50% reduction in coronary risk compared to similarly matched individuals who reported being very sedentary [32]. These results show that regular physical activity not only reduces the risk of developing T2D [33, 34] but also provides clinical benefits among patients with T2D or with the features of the metabolic syndrome. Some studies have used cardiorespiratory fitness (CRF) as an objective physiological marker of participation in vigorous physical activity and have shown that a high level of CRF is associated with a substantially reduced risk of premature mortality, CVD mortality, and CVD morbidity [35–37]. The substantial cardioprotection conferred by a high level of CRF has even been reported among patients with T2D [38]. For instance, Church and colleagues [38] have shown that overweight/obese but fit patients with diabetes were at lower mortality risk than nonobese but unfit patients with diabetes. All the above observations clearly highlight the critical importance of recommending regular physical activity and better cardiorespiratory fitness in patients with T2D. In addition, regular physical activity produces substantial benefits in high-risk individuals with prediabetes, reducing their risk of converting to T2D and developing detrimental cardiovascular outcomes [33, 34].

In addition to aerobic training, moderate-to-high intensity resistance training is beneficial in order to maintain lean body mass, particularly in the aging population of patients with T2D [39–41]. As there is a dose-response relationship between level of physical activity and clinical outcomes, guidelines from the Canadian Society of Exercise Physiology have emphasized the greater health benefits that are expected from a greater volume of weekly physical activity [27]. In line with the Canadian recommendations, it is herein proposed to reduce the time devoted to sedentary behaviour, to increase the level of moderate-to-vigorous physical activities and exercise, and also to perform resistance exercise training for all major muscle groups. Unfortunately, accelerometer data obtained from the 2007–2009 Canadian Health Measures Survey have revealed that only about 15% of Canadian adults accumulate 150 minutes of moderate-to-vigorous physical activity per week, and this statistic is probably even worse among patients with T2D [8]. Because lifestyle modification is a cornerstone of the management of cardiometabolic risk in patients with T2D, it is proposed that efforts and resources should be devoted to help patients afflicted by a societal metabolic disease recalibrate their nutritional and physical activity/exercise habits.

Furthermore, interventions such as motivational interviewing, which is a specific way of helping people recognize and formulate an action plan to address specific lifestyle changes, can be useful for clients who are reluctant or ambivalent about changing behaviour [42]. The strategies used for motivational interviewing are more supportive than confrontational, and the overall goal is to increase a person's intrinsic motivation to change rather than having change imposed by healthcare practitioners [42]. Motivational interviewing, when administered by general practitioners who received training in this treatment modality, has been shown to positively affect attitudes for change in people with T2D [42].

Indeed, it must be remembered that patients find adherence to appropriate dietary patterns exceptionally difficult to maintain consistently and that recommended dietary patterns are not well followed. Furthermore, in the past, diabetes nutrition therapy has emphasized individual macronutrient and micronutrient components and their adequacy. Although studying individual nutrients may lead to an understanding of important biological mechanisms, it has been recognized more recently that providing practical advice or identifing strategies on how people eat is not sufficient. Rather, assessment of dietary patterns offers a comprehensive and complementary approach to apply nutritional principles to “real life” [43] and to identify and validate those that support optimal glycaemic control in people with T2D, regardless of extant pharmacological management. Such assessment has been suggested to be important for advancement of efficacious and effective clinical and public health interventions [43]. Analyses of food patterns would include the possibility that interactions or synergistic effects among individual foods or nutrients are examined [43].

Studies reviewed by Kris-Etherton et al. provide evidence that food-based approaches and dietary patterns reduce risk for cardiovascular disease [44]. For instance, the Breast Cancer Detection Demonstration Project [45] evaluated 42,254 women and demonstrated that all-cause mortality decreased by quartile of recommended food score. The recommended food score was the sum of the number of foods as recommended by current dietary guidelines (fruits, vegetables, whole grains, low-fat dairy and lean meats, and poultry) that were consumed. The age-adjusted relative risk for all-cause mortality in persons in the upper quartile was 0.69 (95% confidence interval 0.61–0.78); for the second and third quartiles the relative risks were 0.82 (95% confidence interval 0.73–0.92) and 0.71 (95% confidence interval 0.62–0.81), respectively. The study demonstrated that as the quality of the dietary pattern improved (on the basis of current dietary guidelines) an associated health benefit was gained. Other reviewed [44] dietary patterns associated with lower or higher risk of chronic disease (resp.) include the Prudent Pattern compared to the Western Pattern, dietary patterns identified in the Nurse's Health Study and the Physician's Health Study. The Prudent Pattern was characterized by a higher intake of vegetables, fruits, legumes, whole grains, and fish while the Western Pattern by a higher intake of processed meat, red meat, butter, high-fat dairy products, eggs, and refined grains. Relative risk for coronary heart disease (CHD) decreased from the lowest to highest quintiles of Prudent Pattern score (relative risk 1.0 and 0.70, 95% confidence interval 0.56–0.86; *P* = 0.0009 for trend), whereas CHD risk increased with increasing quintile for Western Pattern score (relative risk 1.0 and 1.64, 95% confidence interval 1.24–2.17; *P* < 0.0001 for trend). These analyses may provide useful evidence for making specific food-based dietary recommendations within the context of the existing dietary guidelines; however, their impact as part of clinical treatment for diabetes has not been studied.

The Dietary Approaches to Stop Hypertension (DASH) Study demonstrated that a dietary pattern high in fruits, vegetables, and low-fat dairy products, coupled with sodium restriction, reduced hypertension and, consequently, is included here as a dietary intervention for hypertensive patients with T2D (Table 1). Moreover, the Lyon Diet Heart Study showed that dietary patterns have a marked beneficial impact on important risk factors for CVD, as well as morbidity and mortality end-points. These results support the potential positive implications for clinicians and patients of using a dietary pattern approach that emphasizes “what to eat” (i.e., plant-based foods, selected unsaturated fats) rather than “what to restrict” (i.e., total fat, saturated fat, sodium, and sugars) and give more explicit instructions that can be put into practice over the long term. Because evidence continues to emerge about the importance of regularly including particular foods (i.e., nuts, legumes, and vegetables) relative to the risk of developing diabetes and/or other chronic diseases, especially CVD, the Canadian tDNA makes reference to these items (Table 1) in its algorithm. However, background food intake patterns are not usually reported in studies in which one or two foods/nutrients are manipulated, and this information is required in order to understand the potential for interactions between foods and nutrients. Furthermore, it is unlikely that emphasizing a single food or set of foods (e.g., only low GI foods) will significantly and positively impact glycaemic control unless the changes in total intake reflect a significant change in underlying regular dietary patterns [46]. Thus, an emerging issue that must be resolved is that inclusion of particular foods is made within a diet that confers the optimal dietary pattern for risk reduction in a way that promotes a healthy body weight (i.e., it does not exceed energy requirements).

**Figure 3:**
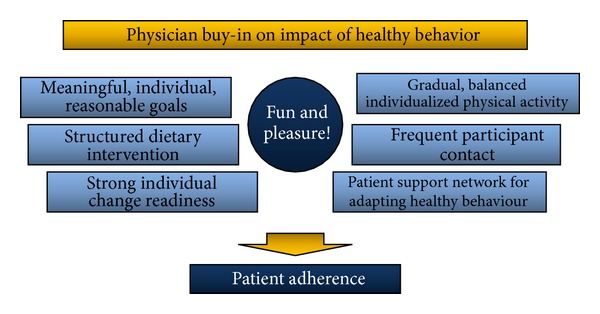
Drivers of adherence for the Canadian tDNA.

Validated evaluation tools and simple/efficient processes for monitoring/surveillance are essential to the success of tDNA. Establishing efficacy is important, but it is also essential to identify techniques, tools, and environmental factors that contributed to, or detracted from, the success of implementing the intervention [47]. Nutrient intake, although important, does not capture the complexity of behavioural changes that people make to implement the dietary advice they received. Details are critical so that programmes can be expanded and adapted when warranted. For the field to advance, we must know what study participants were asked to do and what they actually did and we must move beyond “intention to treat” analyses. Assessment of dietary and physical activity behaviour and behaviour changes requires evaluation tools that are validated, reliable, and easy to use in various clinical practice settings [48]. The tools should be adapted to the cultural specificities of the clients and the Canadian guidelines. An inventory of validated tools, their selection according to accessibility and appropriateness, their adaptation to geographic and ethnocultural specificities, and their modification to improve clarity, simplicity, and user-friendliness remain to be achieved before validation of the tDNA is undertaken in a clinical setting. The tDNA and its tools can only be of use when patients adopt and adhere to the recommendations. This is optimized if the physicians and their patients buy-in on the impact of healthy behaviour on diabetes management and points to the importance of their involvement at each step of the tDNA.  Figure 3 summarizes what is to be accomplished when primary caretakers adopt the Canadian tDNA and its tools in a clinical practice; at its core, we promote to always aim at making the relationship to food and physical activity fun and pleasurable.

## 5. Conclusions

Adapting the global tDNA template to a Canadian society led to the recognition that primary care practitioners need to participate as active and key promoters of healthy lifestyle behaviour with other members of the health professional team. Their involvement entails the development of simple, quick, and effective methods to assess nutritional and physical activity behaviours that put patients at risk and requires the implementation of strategies to help change thi behaviour in a sustainable manner. The foods in the environment of Canadians with T2D should be nutritionally adequate, culturally acceptable through appropriate food and distribution systems, physically and economically accessible at all times, and safe and secure in order to enable adoption of behaviour that promotes optimal diabetes care and make healthy food choices the norm. Furthermore, simple tools should be put in place to first evaluate sedentary behaviour and physical activity habits of patients and then support economically viable solutions to help patients increase their physical activity habits and regular exercise level. Above all, primary care practitioners should buy-in on the impact of healthy lifestyle behaviours on diabetes management by adopting this behaviour themselves. The Canadian tDNA is a first step.

## Figures and Tables

**Table 1:** Dietary strategies for diabetes nutrition therapies.

Interventions	HbA1c %	Wgt	BP	Lipid Risk Factors						Other Advantages	Disadvantage
				LDL-C	Apo-B	HDL-C	TG	Non-HDL-C	Ratio*		
Dietary patterns											
Low-GI/GL	↓ 0.3–0.5%					↑			↓	↓CRP, ↓Hypos, ↓Rx	↓Vitamin B_12_
Veg diets	↓ 0.3–0.5%	↓	*↔*	↓				↓**			
Mediter diets	↓ 0.3–0.5%		↓			↑	↓		↓	↓CRP, ↓FPG, ↓Rx, ↓CV events	
DASH	↓ 0.5–1.0%	↓	↓	↓		↑				↓CRP	
Wgt loss diets											
Atkins	*↔*	↓				↑			↓		↑LDL, ↓micN, ↓adh
Protein power	↓ 0.5–1.0%	↓				↑	↓				↓micN, ↓adh, ↑RL
Omish		↓					↓		↓		*↔*FPG, ↓adh
Wgt watchers		↓							↓		*↔*FPG, ↓adh
Zone		↓							↓		*↔*FPG, ↓adh, ↑RL
Specific foods											
Dietary	↓ 0.3–0.5%			↓							GI side effects
Tree nuts	↓<0.3%			↓	↓		↓		↓		
Macronutrient											
Hi-CHO hi fiber	↓ 0.3–0.5%			↓						Preserve lean mass	↓HDL, GI side effects
Hi-MUFA	↓<0.3%						↓				
Lo-CHO	*↔*						↓				↓micN, ↑RL
Hi-protein	*↔*		↓				↓				↓micN, ↑RL
LC-N3-PUFAs	*↔*		*↔*	*↔*		*↔*	↓				CH_3_–Hg exposure, EI
Meal replacements	↓ 0.3–0.5%	↓									Temporary intervention

Adapted from [4].

Glycaemic index (GI); monounsaturated fatty acids (MUFA); long-chain n-3 polyunsaturated fatty acids (LC-N3-PUFAs); Dietary Approaches to Stop Hypertension (DASH); weight (Wgt); blood pressure (BP); total cholesterol (TC); LDL cholesterol (LDL-C); HDL cholesterol (HDL-C); triglycerides (TG); non-HDL cholesterol (non-HDL-C); apolipoprotein-B (apo-B); fasting plasma glucose (FPG); C reactive protein (CRP); hypos (hypoglycaemic episodes); oral antihyperglycaemic agents (Rx); Mediterranean (Mediter); vegetarian (veg); adherence (adh); micronutrient (micN); renal load (RL); methyl-Hg (M-Hg); environmental impact (EI); gastrointestinal (GI).

*Lipid ratios include TC : HDL-C, LDL-C : HDL-C, and apo-B : apo-A1 (apolipoprotein-A1).

**Adjusted for medication changes.

**Table 2:** Common carbohydrate foods and their glyceamic indices (GI).

Food	GI
Cereals	
Biscuits	69
Cornflakes	81
Instant oatmeal	79
Rice congee	78
Rolled oatmeal	55
Millet porridge	67
Muesli	57
Common items	
Brown rice	68
Barley	28
Chapati	52
Corn	52
Corn tortilla	46
Couscous	65
Multigrain bread	53
Rice noodles	53
Spaghetti	49
Udon noodles	55
Wheat roti	62
White rice	73
White wheat bread	75
Whole wheat bread	74
Dairy products	
Ice cream	51
Skim milk	37
Soy milk	37
Rice milk	86
Whole milk	39
Yogurt	41
Fruits	
Apple	36
Banana	51
Dates	42
Mango	51
Orange	43
Peach	43
Pineapple	59
Watermelon	76
Legumes	
Chickpeas	28
Kidney beans	24
Lentils	32
Soy beans	16
Snacks	
Chocolate	40
Popcorn	65
Potato chips	56
Rice crackers	87
Soda	59
Vegetables	
Potato, boiled	78
Potato, fried	63
Potato, instant mash	87
Sweet potato	63
Carrots, boiled	39
Pumpkin, boiled	64
Plantain	55
Taro, boiled	53
Vegetable soup	48

Glyceamic index (GI) ranks carbohydrates according to their ability to raise blood glucose levels, with the following cut-offs: low-GI ≤ 55, medium-GI 56–69, and high-GI ≥ 70. Adapted from Mechanick et al. [5].

**Table 3:** Canadian Diabetes Association Physical Activity Recommendations for diabetes management.

(1) Patients with diabetes should accumulate a minimum of 150 minutes of moderate-to-vigorous intensity aerobic exercise each week, spread over at least 3 days of the week, with no more than 2 consecutive days without exercise.	
(2) People with diabetes (including elderly people) should also be encouraged to perform resistance exercise 3 times per week, in addition to aerobic exercise. Initial instruction and periodic supervision by an exercise specialist are recommended.	
(3) An exercise ECG stress test should be considered for previously sedentary individuals with diabetes at high risk for CVD who wish to undertake exercise more vigorous than brisk walking (Grade D LOE).	

Adapted from the Canadian Diabetes Association Clinical Practice Guidelines Expert Committee.

Canadian Diabetes Association 2008 clinical practice guidelines for the prevention and management of diabetes in Canada. Can J Diabetes.2008;32 (suppl 1):S1-S201.

**Table 4:** Dietary Approaches to Stop Hypertension (DASH) for diabetes nutrition therapy.

Food groups	Servings per day			Serving size
	1600 kcal/day	2600 kcal/day	3600 kcal/day	
Grains	6	10-11	12-13	1 slice bread; 1 oz dry cereal; 1/2 cup cooked rice, pasta, cereal
Vegetables	3-4	5-6	6	1 cup raw leafy; 1/2 cup cut raw or cooked
Fruits	4	5-6	6	1 medium piece; 1/4 cup dried; 1/2 cup fresh, frozen, canned; 1/2 fruit juice
Low/nonfat dairy	2-3	3	3-4	1 cup milk or yogurt; 1.5 oz cheese
Lean meat, poultry, and fish	3–6	6	6–9	1 oz cooked, meats, fish; 1 egg
Nuts, seeds, and legumes	3/week	1	1	1/3 cup nuts; 2 tbsp peanut butter; 2 tbsp seeds; 1/2 cup cooked legumes
Fats and Oils	2	3	4	1 tsp soft margarine (nonhydrogenated); 1 tsp veg oil; 1 tbsp mayonnaise; 2 tbsp salad dressing
Sweets, added sugars	0	≤2	≤2	1 tbsp sugar; 1 tbsp jelly or jam; 1/2 cup sorbet, gelatin; 1 cup lemonade

Adapted from the Canadian Diabetes Association.

Canadian Diabetes Association, DASH diet summary, accessed at http://www.diabetes.ca/documents/about-diabetes/DASH_Diet_Summary.pdf on 11, 01, 2012.

**Table 5:** CDA's Clinical Practice Guidelines Suggestions for bariatric surgery.

(1) Adults with clinically severe obesity (BMI ≥ 40 kg/m^2^ or ≥35 kg/m^2^ with severe comorbid disease) may be considered for bariatric surgery when lifestyle intervention is inadequate to achieve healthy weight goals.	
(2) Bariatric surgery in adolescents should be limited to exceptional cases and performed only by experienced teams.	
(3) A minimally invasive approach should be considered for weight loss surgery when an appropriately trained surgical team and appropriate resources are available in the operating theatre.	
